# The effects of low-load resistance training combined with blood flow restriction on knee rehabilitation in middle-aged and elderly patients: A systematic review and meta-analysis

**DOI:** 10.1371/journal.pone.0323388

**Published:** 2025-06-02

**Authors:** Juan Chen, Lei Wu, Chenna Li, Hao Yan

**Affiliations:** 1 School of Rehabilitation Engineering, China Civil Affairs University, Beijing, China; 2 School of Strength and Conditioning Training, Beijing Sport University, Beijing, China; Universiti Malaya, MALAYSIA

## Abstract

This meta-analysis evaluates the effectiveness of low-load resistance training combined with blood flow restriction in knee rehabilitation. Methods: Randomized controlled trials investigating the effects of blood flow restriction training on knee injury rehabilitation were systematically searched in the PubMed, EBSCO, and Web of Science databases for studies published between January 2000 and May 2024. The Cochrane Risk of Bias Tool was used to assess study quality, and statistical analyses were performed using Review Manager 5.3 software. Results: (1) Compared to low-load control training, blood flow restriction training showed no significant difference in pain scores (standardized mean difference = -0.10, P = 0.46) but significantly improved muscle strength (standardized mean difference = 1.11, P < 0.00001). (2) When compared to high-intensity resistance training, blood flow restriction training demonstrated no significant differences in muscle strength (standardized mean difference = -0.11, P = 0.74) or pain scores (standardized mean difference = -0.84, P = 0.17). (3) Preoperative blood flow restriction training did not significantly improve postoperative pain scores (standardized mean difference = 0.77, P = 0.37); however, among 241 patients undergoing preoperative training, blood flow restriction training significantly enhanced postoperative muscle strength (standardized mean difference = 0.97, P = 0.03). Conclusions: Although blood flow restriction training has limited effects on reducing pain, it significantly improves muscle strength, particularly in preoperative rehabilitation and low-load training settings, making it a valuable alternative in clinical knee rehabilitation strategies.

## Introduction

Knee joint disorders, including knee osteoarthritis (KOA) [1], total knee arthroplasty (TKA) [2], cartilage injuries [3], and ligament injuries [4,5], have become significant health concerns among middle-aged and elderly populations worldwide. Global statistics reveal that approximately 25% of adults suffer from knee joint issues [6], with prevalence rates rising sharply among older age groups [7]. As individuals age, knee-related conditions contribute to pain, functional limitations, and reduced mobility, significantly impairing quality of life [8]. Traditional resistance training is commonly employed to enhance muscle strength [9,10]; however, high-intensity regimens often impose excessive strain on the joints, making them unsuitable for many patients with knee injuries. Pain and discomfort frequently prevent patients from tolerating such intense exercise, thereby limiting its therapeutic effectiveness [11]. This highlights the urgent need for an alternative rehabilitation approach that can simultaneously restore muscle strength and manage pain more effectively.

Blood flow restriction (BFR) training, a low-intensity exercise modality, has gained significant attention in recent years for its application in healthy individuals [12] and athletes [13]. By applying external pressure to limit venous return while maintaining arterial inflow, BFR training creates a hypoxic environment that induces metabolic stress, thereby promoting muscle hypertrophy and strength gains [14–16]. Research suggests that BFR training can achieve muscle growth comparable to high-intensity training while minimizing joint stress, making it an appealing option for patients with knee injuries, particularly in post-operative rehabilitation [17,18]. Given its ability to improve muscle strength while reducing joint strain, BFR training has been increasingly recognized as a promising rehabilitation strategy for this population.

However, despite its growing recognition, the effectiveness of BFR training in pain management remains controversial. Some studies have reported that BFR training alleviates pain by promoting endogenous opioid release and reducing inflammation [19,20], while others have found no significant pain relief effects, particularly in older individuals with chronic knee conditions [21,22]. This lack of consensus on BFR’s pain-modulating effects creates uncertainty regarding its clinical applicability in knee rehabilitation. Moreover, most existing research focuses on the safety, efficacy, and post-operative benefits of BFR training, with limited exploration of its role in pre-operative rehabilitation or its effects on post-operative pain and muscle recovery in older adults. These gaps highlight the need for a systematic evaluation of BFR training’s impact on both muscle strength and pain outcomes in knee rehabilitation.

This study aims to systematically evaluate the rehabilitation potential of low-load resistance training combined with BFR (LLRT-BFR) in middle-aged and elderly patients with knee injuries. The specific objectives are: (1) to assess the effects of LLRT-BFR compared to conventional rehabilitation methods on muscle strength and pain; (2) to compare the efficacy of LLRT-BFR with high-intensity training in terms of muscle strength recovery and pain management; (3) to investigate the impact of pre-operative LLRT-BFR on post-operative muscle strength and pain outcomes.

## Methods

### Literature search

Two researchers independently conducted a systematic literature search across seven electronic databases: Web of Science, PubMed, EBSCO, Cochrane Library, Embase, Scopus, and China National Knowledge Infrastructure (CNKI). Additionally, ClinicalTrials.gov was searched for relevant registered trials. The search covered articles published from January 2000 to the final search date, November 16, 2024. The search strategy included the following English terms: Kaatsu, Blood Flow, BFR, ACL, Occlusion, Osteoarthritis, Arthritis, KOA, OA, Knee, Exercise, and Training. The Boolean search formula applied was: (“Kaatsu” OR “Blood Flow Restriction” OR “BFR”) AND (“ACL” OR “Knee” OR “Osteoarthritis” OR “Arthritis” OR “KOA”) AND (“Exercise” OR “Training”). During the search process, two researchers independently screened article titles and abstracts for relevance. If any information was unclear, the corresponding authors were contacted via email for clarification. In cases of disagreement, a third researcher was consulted to facilitate discussion and reach a consensus.

This systematic review and meta-analysis was prospectively registered in the PROSPERO database with the registration number CRD42046892642. The review protocol adheres to the Preferred Reporting Items for Systematic Reviews and Meta-Analyses guidelines.

### Inclusion and exclusion criteria

#### Inclusion criteria.

The study subjects must be patients with knee injuries who underwent blood flow restriction (BFR) training as an intervention, with the intervention lasting at least 2 days. The method of BFR training (such as device type, pressure, frequency, etc.) must be clearly described in the literature. The outcome measures must include at least muscle strength or pain scores, or functional recovery-related indicators. Additionally, the study subjects should be middle-aged and older adults with knee injuries (≥45 years), with both the experimental and control groups consisting of knee injury patients who have no severe comorbidities or other diseases that may affect rehabilitation (such as cardiovascular or neurological disorders). Only studies published in English or Chinese will be included, and non-English or non-Chinese studies must provide a reliable translation.

#### Exclusion criteria.

**Fig 1:**
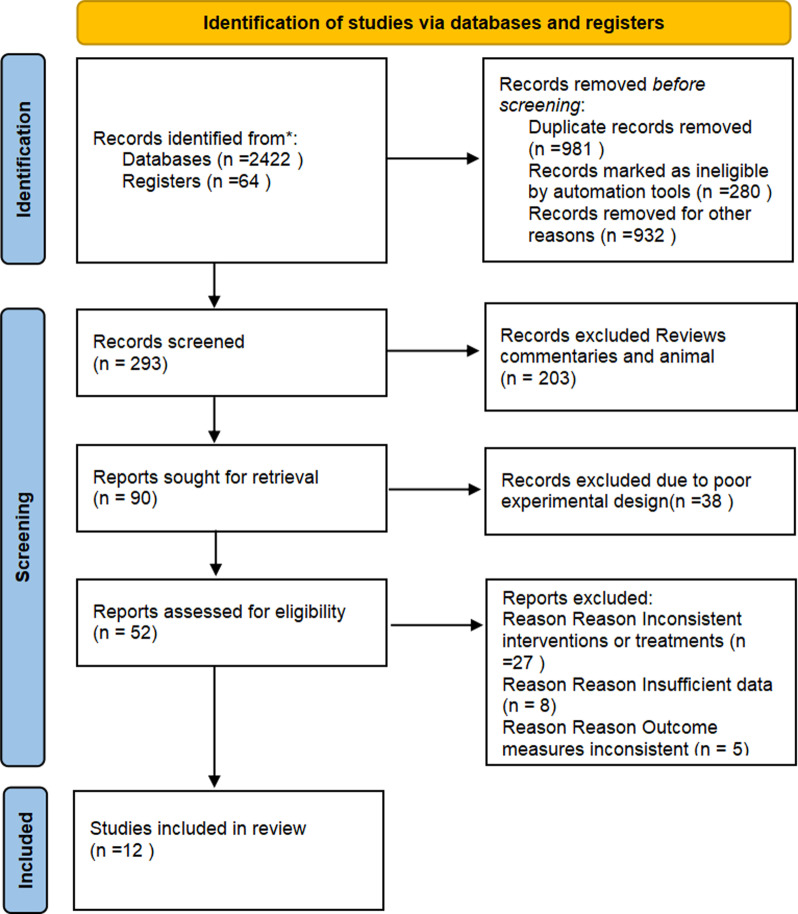
Flowchart of Literature Screening.

Exclusion criteria include: duplicate studies; review articles, systematic reviews, meta-analyses, or animal studies; non-English or non-Chinese literature without a reliable translation; non-full-text articles that could not be obtained via email contact; studies with inadequate experimental design, lacking randomized control, or with outcome measures that do not meet the inclusion criteria; studies with subjects under 45 years of age or with severe comorbidities or confounding factors; studies with a small sample size, significant data loss, or poor quality (Fig 1).

#### Data extraction.

Two researchers independently and blindly extracted data from the selected studies. The extracted information included: the first author and publication year, participant gender, age, sample size, intervention details, and outcome measures.

### Quality assessment of studies

**Fig 2:**
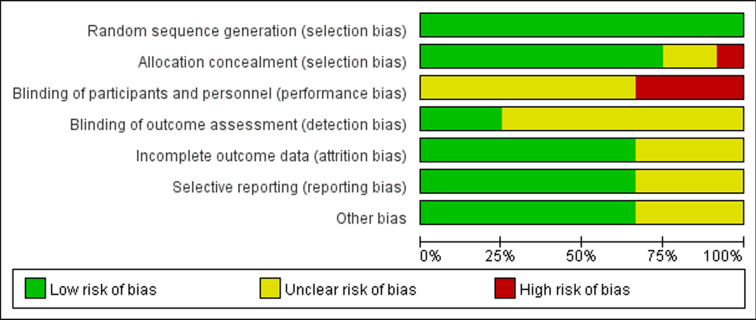
Risk of Bias Plot.

**Fig 3:**
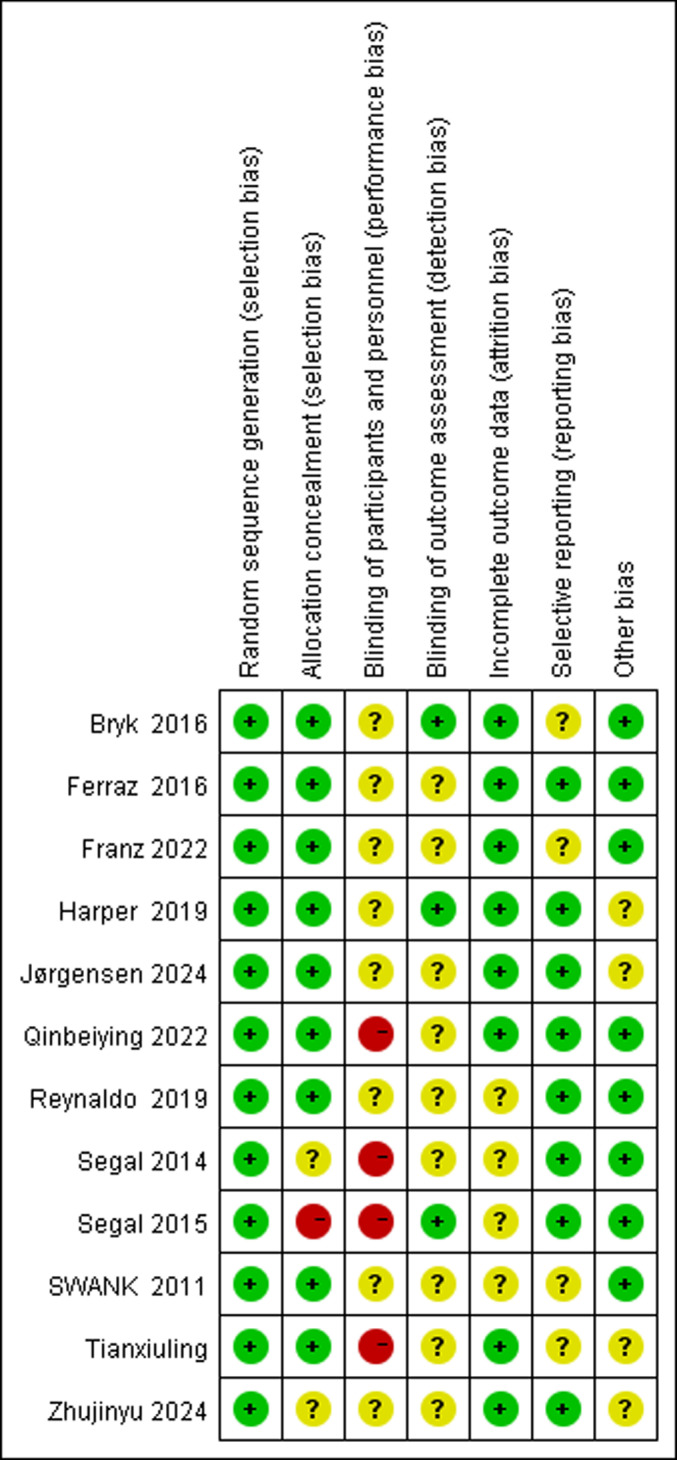
Summary of Risk of Bias.

The quality of the included studies was assessed using the Cochrane Risk of Bias Tool [22]. The evaluation focused on the following aspects: random sequence generation, allocation concealment, blinding of participants and researchers, blinding of outcome assessment, incomplete outcome data, selective reporting, and other potential biases. Each item was rated as “Yes” (low risk of bias), “No” (high risk of bias), or “Unclear” (unable to determine risk of bias). Fig 2 presents the distribution of risk of bias across studies for each item, and Fig 3 summarizes the overall risk of bias.

### Assessment of publication bias

Funnel plots were used to visually assess potential publication bias among the included studies. To quantitatively evaluate the symmetry of the funnel plot, Egger’s regression test was performed. Egger’s test assesses whether there is a statistically significant asymmetry in the distribution of effect sizes, which may indicate publication bias. If publication bias was detected, sensitivity analyses were conducted to assess its potential impact on the robustness of the findings.

### Data analysis

Review Manager 5.3.5 software was used for the analysis. The standardized mean difference (SMD) was chosen as the effect size due to variations in measurement across studies [23]. Heterogeneity was assessed using the I² statistic: a fixed-effects model was applied if I² < 50%, and a random-effects model if I² ≥ 50% [24]. Subgroup analysis explored sources of heterogeneity, and funnel plots assessed potential publication bias.

## Results

### Basic characteristics of included studies

A total of 12 randomized controlled trials (RCTs) were included in this study[25–36], involving 642 middle-aged and elderly patients, comprising 193 men and 378 women. Among them, 120 participants were assigned to the preoperative low-load blood flow restriction (L-BFR) group, 192 to the postoperative L-BFR group, 285 to the low-load resistance training (L-CON) group, and 45 to the high-load resistance training (H-RT) group.Eight studies reported lower limb extensor strength as an outcome measure [24–30,32], while 10 studies included physical function tests as outcome variables [25–27,29,30,32–36], and all 12 studies measured pain scores as an outcome [25–36]. The primary condition was osteoarthritis in eight studies [25–30,32,33], and total knee arthroplasty (TKA) in five studies [31,33–36].

### Variability in intervention protocols

The included studies exhibited notable variability in training protocols, particularly in training duration, occlusion pressure, and exercise intensity. Preoperative interventions ranged from 3 days to 8 weeks, while postoperative interventions lasted 6–14 weeks. Training was typically performed 2 to 5 days per week, with 4–6 sets per session and 10–30 repetitions per set, although some studies implemented progressive overload, while others maintained a fixed workload.

Occlusion pressure settings varied, with some studies using 40–60% limb occlusion pressure (LOP), while others applied absolute pressures ranging from 97.4 to 200 mmHg. Most studies used continuous occlusion, while some incorporated intermittent occlusion release. Sham BFR conditions were inconsistent, with some studies applying 20 mmHg, while others did not specify sham settings.

Training intensity also differed, with most studies prescribing low-load resistance training (20 ～ 30% 1RM), whereas high-load resistance training groups used 60–80% 1RM. Exercise selection included leg press, knee extension, and calf flexion, with variations in single-joint vs. multi-joint exercises. Rest intervals ranged from 30 to 180 seconds, influencing metabolic stress and neuromuscular recovery.

These differences in training parameters may have contributed to the heterogeneity in study outcomes, highlighting the need for standardized intervention protocols in future research (Table 1).

**Table 1:** Summary of Basic Characteristics of Included Studies.

Author	Year	Disease Type	Sample Size, Gender, Age	Duration (weeks)	Training Method	Outcome Measures
Segal et al. [27]	2014	OA	L-BFR: 19 M, 58.4 ± 8.7 yrs; L-CON: 22 M, 56.1 ± 7.7 yrs	4	L-BFR and L-CON, leg press: 1 set of 30 reps, 3 sets of 15 reps, 3 days/wk	MLES, SCMP, KOOS
Segal et al. [28]	2015	OA	L-BFR: 19 F, 56.1 ± 5.9 yrs; L-CON: 21 F, 54.6 ± 6.9 yrs	4	L-BFR and L-CON, leg press: 1 set of 30 reps, 3 sets of 15 reps, 3 days/wk	MLES, KOOS
Harper et al. [29]	2019	OA	L-BFR: 6 M, 10 F, 67.2 ± 5.2 yrs; H-RT: 15 F, 4 M, 69.1 ± 7.1 yrs	12	L-BFR: 20% 1RM; H-RT: 60% 1RM, leg press, leg ext, calf flexion, leg curl, 3 days/wk	MLES, 400m walk, WOMAC
Ferraz et al. [25]	2016	OA	L-BFR: 12 F, 60.3 ± 3.0 yrs; H-RT: 10 F, 59.9 ± 4.0 yrs; L-CON: 12 F, 60.7 ± 4.0 yrs	12	L-BFR and L-CON: 30% 1RM; H-RT: 80% 1RM, 4–5 sets of 10 reps, 2 days/wk	MLES, TUG, WOMAC
Bryk et al. [26]	2016	OA	L-BFR: 17 F, 62.3 ± 7.0 yrs; L-CON: 17 F, 60.4 ± 6.7 yrs	6	L-BFR: 30% 1RM, knee ext, 3 sets of 30 reps; H-RT: 70% 1RM, 3 sets of 10 reps, 3 days/wk	MLES, TUG, NPRS
Reynaldo et al. [30]	2019	RA	L-BFR: 16 F, 59.6 ± 3.9 yrs; H-RT: 16 F, 58.0 ± 6.6 yrs	12	L-BFR and H-RT: bilateral leg press; L-BFR: 4 sets of 15 reps; H-RT: 70% 1RM, 4 sets of 10 reps, 2 days/wk	MLES, TUG, SF-36
Tianxiuling et al. [31]	2021	KR	L-BFR: 22 M, 28 F, 62.76 ± 8.14 yrs; L-CON: 24 M, 26 F, 63.21 ± 7.53 yrs	14	L-BFR and L-CON, leg press: 4 sets of 15 reps, 3 days/wk	PTKE
Qinbeiying et al. [32]	2022	OA	L-BFR: 19 M, 24 F, 66.33 ± 3.36 yrs; L-CON: 16 M, 26 F, 67.24 ± 4.11 yrs	8	L-BFR and L-CON: 20–30% 1RM, knee ext, 5 sets of 10 reps, 5 days/wk	MLES, GS, WOMAC, VAS
Swank et al. [33]	2011	OA	L-BFR: 36, 63.1 ± 7.3 yrs; L-CON: 35, 62.6 ± 7.6 yrs	8	L-BFR and L-CON: 1RM, leg press, 3 days/wk	PT, 6MWD, VAS
Zhujinyu et al. [34]	2024	KR	L-BFR: 4 M, 28 F, 64.97 ± 5.78 yrs; L-CON: 6 M, 26 F, 65.16 ± 8.12 yrs	Pre-op 3 days	L-BFR and L-CON: 30% 1RM, knee ext, 4 sets	QS, FAT
Franz et al. [35]	2022	KR	L-BFR: 7 M, 3 F, 61.5 ± 8.8 yrs; L-CON: 7 M, 3 F, 64.2 ± 7.7 yrs	6	L-BFR and L-CON: 6RM, knee ext	6RM, 6MWT, KOOS
Jørgensen et al. [36]	2024	KR	L-BFR: 16 M, 26 F, 67.1 ± 8.18 yrs; L-CON: 21 M, 23 F, 66.1 ± 7.73 yrs	8	L-BFR and L-CON: leg press, knee ext, 4 sets of 30 reps, 3 days/wk	KOOS, TUG, MLES

Note: OA: Osteoarthritis; RA: Rheumatoid Arthritis; KR: Knee Replacement; L-BFR: Low-load Blood Flow Restriction; L-CON: Low-load Control; H-RT: High-intensity Resistance Training; 1RM: One Repetition Maximum; 6RM: Six Repetition Maximum; 6MWD: 6-Minute Walk Distance; MLES: Maximum Leg Extensor Strength; PT: Peak Torque; TUG: Timed Up and Go Test; KOOS: Knee Injury and Osteoarthritis Outcome Score; SCMP: Self-Care Mobility Profile; WOMAC: Western Ontario and McMaster Universities Osteoarthritis Index; VAS: Visual Analog Scale; PTKE: Peak Torque of Knee Extensors; GS: Gait Speed; QS: Quadriceps Strength; FAT: First Ambulation Time; SF-36: Short Form (36) Health Survey

### Sensitivity analysis

Sensitivity analysis was performed by altering the analytical model, selecting different effect sizes, and excluding individual studies. The results of the meta-analysis remained consistent after sensitivity testing, confirming the robustness of the findings.

### Publication bias

**Fig 4:**
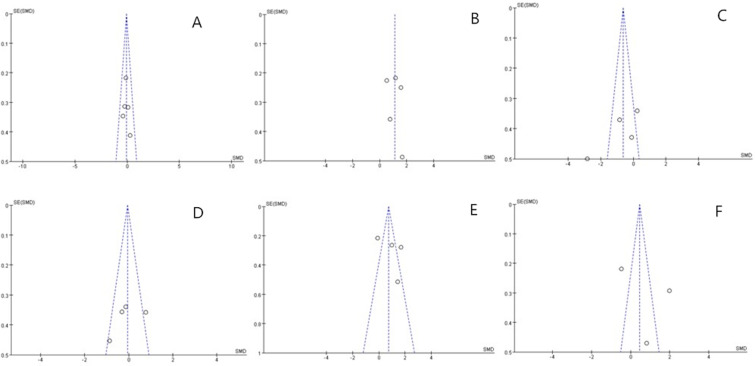
Funnel Plot.

This analysis included 12 studies, meeting the minimum sample size required for funnel plot construction, as supported by Lu et al. [37]. Funnel plots of the included studies are presented in Fig 4A–4F. Notably, Fig 4C and 4D display apparent asymmetry, suggesting the presence of potential publication bias. This may indicate that studies with significant findings are more likely to be published, thereby inflating the overall effect size. Contributing factors may include the inclusion of low-quality studies or variations in BFR training protocols (e.g., frequency, intensity, duration).

### Meta-analysis results

#### L-BFR vs. L-CON on pain scores.

**Fig 5:**
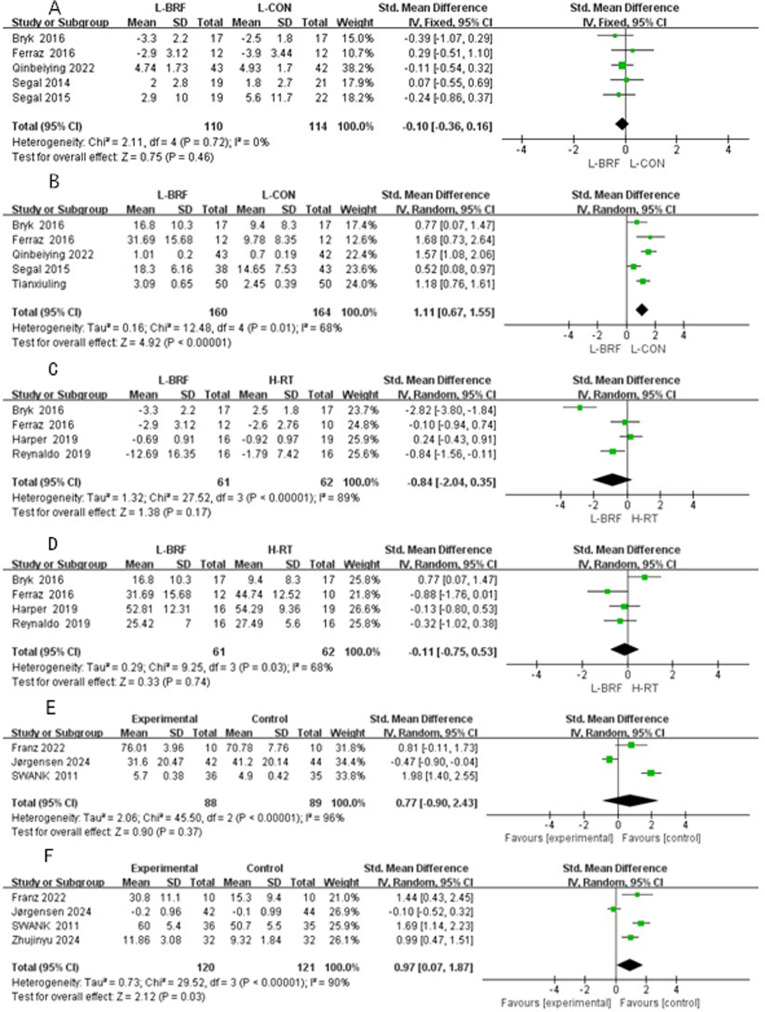
A-F Forest Plot.

A meta-analysis was conducted on 5 studies, including 224 participants, to evaluate the overall effect of L-BFR training compared to L-CON on pain scores (Fig 5A). The results showed an effect size of SMD = -0.10 (95% CI: -0.36 to 0.16; Z = 0.75; P = 0.46), indicating no significant difference in pain scores between L-BFR (n = 110) and L-CON (n = 114) groups (P = 0.46). As there was homogeneity among the studies (I² = 0, P = 0.72), a fixed-effects model was used.

#### L-BFR vs. L-CON on muscle strength.

A meta-analysis of 5 studies involving 324 participants was performed to evaluate the impact of L-BFR compared to L-CON on muscle strength (Fig 5B). The results revealed an effect size of SMD = 1.11 (95% CI: 0.67 to 1.55; Z = 4.92; P < 0.00001), demonstrating that L-BFR training significantly improved muscle strength compared to L-CON. Due to heterogeneity among the studies (I² = 68%, P = 0.01), a random-effects model was applied. Subgroup analysis by gender revealed homogeneity within subgroups (I² = 0), indicating that gender was a source of heterogeneity (Table 2). The effect size for the female group was SMD = 0.77 (95% CI: 0.07 to 1.47; P = 0.002), while for the mixed group, it was SMD = 1.68 (95% CI: 0.73 to 2.64; P < 0.00001), suggesting that L-BFR improved muscle strength across different genders.

**Table 2:** Subgroup analysis.

Group Classification	Heterogeneity Test			Subgroup	Size (95% CI)	Z-Test		Studies	Sample Size
	I^2^		P		Z	P			
Pain BFR VS CON	68	0	0.66	Female	0.77 [0.07, 1.47]	3.1	0.002	3	160
		0	0.41	Mixed	1.68 [0.73, 2.64]	8.32	< 0.00001	4	164
Pain BFR VS HRT	89	NA		70%1RM	-2.82 [-3.80, -1.84]	5.64	< 0.00001	1	17
		57	0.10	60-80%1RM	-0.23 [-1.68, 1.22]	0.68	0.50	3	44
Strength BFR VS HRT	68	NA		70%1RM	0.77 [0.07, 1.47]	2.16	0.03	1	17
		0	0.42	60-80%1RM	-0.37 [-0.79, 0.05]	1.72	0.09	3	44
Pre-op Strength BFR VS CON	90	40	0.19	low load	1.35 [0.87, 1.83]	5.5	0.00001	3	78
		NA		Usual care	-0.10 [-0.52, 0.32]	0.47	0.64	1	42

Note: BFR: Blood Flow Restriction; CON: Control; HRT: High Resistance Training; 1RM: One Repetition Maximum; NA: Not Applicable.

#### L-BFR vs. H-RT on muscle strength and pain scores.

Four studies with a total of 123 participants were included to compare the effects of L-BFR (n = 61) and H-RT (n = 62) on muscle strength (Fig 5C) and pain scores (Fig 5D). The results showed no significant difference in muscle strength between the two groups (SMD = -0.11, 95% CI: -0.75 to 0.53; Z = 1.38; P = 0.74). Due to heterogeneity among studies (I² = 68%, P = 0.03), a random-effects model was used. Subgroup analysis based on training intensity revealed homogeneity within subgroups (I² = 0), indicating that training intensity was a source of heterogeneity (Table 2). Regarding pain scores, no significant difference was observed between the groups (SMD = -0.84, 95% CI: -2.04 to 0.35; P = 0.17). Due to high heterogeneity (I² = 89%, P < 0.00001), a random-effects model was employed. Subgroup analysis by training intensity revealed that the 60–80% 1RM group reduced heterogeneity to a moderate level, indicating that training intensity was a source of heterogeneity (Table 2). The effect size for the 70% 1RM group was SMD = -2.82 (95% CI: -3.80 to -1.84; P = 0.17), but this was based on only one study and lacked representativeness. The effect size for the 60–80% 1RM group was SMD = -0.23 (95% CI: -1.68 to 1.22; P = 0.50).

#### Pre- and postoperative BFR training on pain scores.

Three studies with a total of 177 participants were analyzed to compare pre- and postoperative BFR training with controls on pain scores (Fig 5E). The results showed no significant difference between L-BFR (n = 88) and L-CON (n = 89) groups (SMD = 0.77, 95% CI: -0.90 to 2.43; Z = 0.90; P = 0.37). Due to high heterogeneity among studies (I² = 96%, P < 0.00001), a random-effects model was used.

#### Preoperative BFR training on postoperative muscle strength.

Four studies with a total of 241 participants were analyzed to evaluate the impact of preoperative BFR training on postoperative muscle strength compared to controls (Fig 5F). The results showed a significant difference in muscle strength between the L-BFR group (n = 120) and the L-CON group (n = 121) (SMD = 0.97, 95% CI: 0.07 to 1.87; Z = 2.13; P = 0.03). Due to high heterogeneity among studies (I² = 90%, P < 0.00001), a random-effects model was applied. Subgroup analysis by rehabilitation methods reduced heterogeneity to a low level, indicating that rehabilitation methods were a source of heterogeneity (Table 2). The effect size for the low-load group was SMD = 1.35 (95% CI: 0.87 to 1.83; P < 0.00001), while for the usual care treatment group, it was SMD = -0.10 (95% CI: -0.52 to 0.32; P = 0.64). These findings suggest that preoperative low-load BFR training significantly improves postoperative strength, while usual care treatment has no significant effect.

## Discussion

### The effects of BFR training on muscle strength in patients with knee injuries and its physiological mechanisms

L-BFR training is widely used to enhance muscle strength in athletes and older adults, and its application in rehabilitation has expanded [38,39]. Studies consistently show that BFR training significantly improves strength [40–42]. Hughes et al. found that L-BFR training outperformed traditional low-load training in musculoskeletal rehabilitation but was less effective than high-load training [40]. Rodrigo-Mallorca et al. reported significant strength gains (SMD = 0.61) in older adults, comparable to high-intensity training [41]. Wengle et al. found postoperative BFR training enhanced quadriceps strength and reduced muscle atrophy, but preoperative BFR showed no effect [42].

Our study aligns with these findings, confirming that L-BFR training is more effective than traditional L-CON in improving muscle strength in patients with knee injuries. Both L-BFR and H-RT yielded significant strength improvements, yet L-BFR provided superior outcomes compared to L-CON. Interestingly, we observed that preoperative L-BFR training significantly improved postoperative muscle strength, contrary to Wengle et al.‘s results [42]. The discrepancies between studies could be attributed to variations in training protocols (such as cuff pressure, training intensity, and duration), patient characteristics (such as baseline muscle strength or preoperative atrophy), and the methods used to assess muscle strength (subjective vs. objective measures). Additionally, differences in the interventions provided to control groups may contribute to the differences observed in outcomes, with our control group potentially receiving less structured rehabilitation compared to that of Wengle et al.’s study, which could influence the results.

BFR training enhances muscle strength through several key physiological mechanisms, including metabolic stress, neuromuscular adaptation, endocrine responses, and cellular swelling. The cumulative effects of these mechanisms help stimulate muscle hypertrophy and strength development, even under low external loads.

### Metabolic stress and lactate accumulation

BFR training creates a hypoxic and ischemic environment in the target muscles by partially restricting venous return while maintaining arterial inflow. This condition reduces metabolite clearance, leading to the accumulation of lactate, hydrogen ions, and inorganic phosphate [43,44]. The buildup of these metabolic by-products increases intramuscular acidosis, which plays a critical role in initiating anabolic responses.

The metabolic stress induced by BFR training triggers a hormonal cascade, stimulating the secretion of growth hormone (GH), insulin-like growth factor-1 (IGF-1), and testosterone—all of which are essential for muscle protein synthesis and hypertrophy [45,46]. Additionally, elevated lactate levels enhance motor unit recruitment, particularly in fast-twitch (Type II) muscle fibers, which are responsible for high force output [47]. This preferential activation of Type II fibers under low-load conditions is a key factor distinguishing BFR training from conventional low-load resistance training.

### Neuromuscular adaptation and fast-twitch fiber recruitment

Under normal conditions, low-load resistance training primarily activates slow-twitch (Type I) muscle fibers, as these fibers rely on oxidative metabolism. However, during BFR training, the restricted oxygen supply forces the recruitment of fast-twitch (Type II) fibers to compensate for the increased metabolic demand. This shift in fiber recruitment is crucial for enhancing neuromuscular adaptations and promoting greater force generation.

The increased activation of high-threshold motor units during BFR training leads to significant neuromuscular adaptations, even at low external loads [48]. Electromyographic (EMG) studies have demonstrated that muscle activation during low-load BFR training is comparable to that observed in high-resistance training (H-RT), reinforcing the effectiveness of BFR for strength development [49]. This finding supports the notion that BFR training can elicit substantial neural drive and motor unit engagement, facilitating muscle strength improvements without the need for high mechanical loads.

### Endocrine and molecular signaling pathways

The combination of hypoxia and metabolic stress induced by BFR training stimulates multiple anabolic signaling pathways, which promote muscle hypertrophy and protein synthesis. One of the most critical pathways is the mechanistic target of rapamycin (mTOR) signaling cascade, which regulates muscle protein synthesis and adaptation. Studies have shown that BFR training significantly upregulates mTOR activity, thereby enhancing muscle growth and strength [50].Additionally, systemic increases in GH and testosterone following BFR training further amplify muscle hypertrophy and strength gains [51]. These hormonal responses are comparable to those observed in high-intensity resistance training, suggesting that BFR training can mimic the endocrine effects of traditional strength training despite using lower external loads.

### Cellular swelling and anabolic response

BFR training induces cellular swelling due to venous pooling in the restricted limb, which creates an osmotic shift that draws fluid into muscle cells. This increase in intracellular pressure serves as a potent anabolic stimulus, promoting protein synthesis while inhibiting protein degradation [52]. The mechanosensitive pathways activated by this swelling response further contribute to muscle hypertrophy and structural adaptations [53]. The combination of cellular swelling, metabolic stress, and endocrine signaling reinforces BFR training’s ability to induce muscle hypertrophy with minimal mechanical load, making it an effective rehabilitation tool.

### BFR training’s effects on pain in patients with knee injuries

While BFR training is highly effective in enhancing muscle strength, its impact on pain relief is less clear. Several studies have shown that BFR training can reduce pain in certain patient populations, but the findings remain inconsistent. Li et al. [38] found that in rehabilitation training for patients with knee injuries, the L-BFR group exhibited significant reductions in pain scores, indicating an advantage in alleviating pain. Conversely, Cuyul-Vásquez et al conducted a systematic review focusing on patients with knee pain and concluded that incorporating BFR into resistance training did not significantly reduce pain scores, suggesting limited short-term effects [41]. Similarly, Nitzsche et al analyzed patients with lower-limb musculoskeletal disorders and found no significant difference between L-BFR and traditional resistance training in pain relief [42].

The present study compared results from three perspectives: L-BFR versus L-CON, L-BFR versus H-RT, and preoperative L-BFR intervention versus conventional care. Across these comparisons, L-BFR showed limited effects, further demonstrating that its use does not significantly alleviate patient pain. This finding contrasts with previous studies that suggested BFR training has analgesic effects, potentially reducing knee pain before surgery, immediately after surgery, and up to 24 hours postoperatively [38]. Although BFR demonstrates significant efficacy in improving muscle strength and promoting functional recovery, its effectiveness in pain relief may be limited or even counterproductive in certain cases.

From a mechanistic perspective, BFR’s analgesic effects primarily rely on metabolic pressure-induced endogenous opioid release and inflammation modulation [51]. However, these mechanisms may only apply to inflammatory pain [52]. For mechanical pain (e.g., pain caused by joint lesions or soft tissue injuries), neuropathic pain, or pain associated with central sensitization, BFR’s effects may be limited. For instance, in chronic pain patients, central sensitization can cause persistent pain signaling even after peripheral pain sources have been addressed, potentially hindering pain relief [53].

Additionally, BFR application may have adverse effects. The accumulation of lactate and local hypoxia may induce metabolic pain, while cuff compression could directly stimulate the skin and nerve endings, causing mechanical discomfort [54]. Such adverse effects may obscure or counteract BFR’s potential analgesic benefits. Pain is also a complex, multidimensional phenomenon involving physiological, psychological, and emotional factors. For some patients, BFR might induce anxiety or exacerbate pain perception, weakening its analgesic efficacy.

Design flaws in interventions may further limit BFR’s effectiveness. For example, setting cuff pressure too high or too low could impact results [55]. Short intervention durations or inappropriate timing of pain measurement might miss the peak manifestation of analgesic effects. Existing literature also supports this view, as studies by Cuyul-Vásquez et al. [41] and Nitzsche et al. [42] failed to find significant pain relief effects of BFR for knee or other lower-limb pain, contradicting traditional assumptions about BFR’s analgesic mechanisms. These findings suggest that BFR may not be suitable for all types of pain or patient populations. Its application in pain management should be carefully tailored to individual cases.

Overall, the variability in BFR’s effects on pain may be attributed to differences in pain types, patient characteristics, training parameters, and psychological factors. These limitations necessitate further research into the underlying mechanisms and optimization of intervention strategies to clarify its scope of application and best practices.

### Clinical implications and heterogeneity in BFR training application

While BFR training has demonstrated effectiveness in muscle strength enhancement, its impact on pain relief is variable and influenced by a variety of factors. In clinical practice, the heterogeneity of patient populations, training protocols, and equipment used can significantly affect the outcomes of BFR training. It is essential to consider individual patient characteristics—such as baseline muscle strength, injury severity, rehabilitation phase, and pain type—when designing BFR interventions. Patient-specific modifications to BFR protocols, such as adjusting cuff pressures, training intensity, or session frequency, may be necessary to optimize the benefits while minimizing potential risks. For example, older patients or those with severe muscle atrophy may benefit from lower cuff pressures and less frequent sessions, while athletes or younger patients may require more intense protocols to maximize strength gains. Additionally, variations in the equipment used (e.g., cuff design, inflation systems) and inconsistencies in training protocols may contribute to the heterogeneous effects of BFR training. To ensure safe and effective treatment, clinicians should regularly monitor patients and adjust BFR parameters as needed. Personalized assessments of muscle strength and pain should guide the modification of training protocols, with careful consideration of the patient’s progress, subjective reports, and objective outcomes. Further research on patient-tailored BFR applications will be invaluable in establishing more specific and universally applicable guidelines for its use in clinical settings.

## Conclusion

While L-BFR has shown limited effects on pain relief, it demonstrates significant benefits in enhancing muscle strength, making it a valuable option for preoperative rehabilitation and low-load training environments. These strengths highlight its potential as an effective rehabilitation tool, particularly in clinical settings. However, to enhance its generalizability and optimize its clinical application, further research is needed to establish standardized protocols and conduct large-scale trials. Such efforts will help validate the long-term effects of L-BFR and ensure its applicability across diverse patient populations and injury types.

## Limitations and future directions

The studies included in this meta-analysis generally had small to moderate sample sizes, which may increase the risk of Type I errors and limit the robustness of the results. Additionally, inconsistencies in cuff pressure quantification for BFR training hinder direct comparisons across studies and contribute to heterogeneity. This analysis could not establish a dose-response relationship, as existing literature provides insufficient exploration of the mechanisms behind pressure, training frequency, and intensity. Given the limited number of related studies, the generalizability and representativeness of this study’s findings need to be validated through larger sample sizes and standardized RCTs.

Future research should prioritize establishing standardized cuff pressure quantification protocols to reduce heterogeneity across studies and enhance the comparability and clinical relevance of findings. Further exploration of the dose-response relationship is needed to identify the specific impacts of pressure levels, training frequency, and intensity on pain relief and muscle strength enhancement, thereby optimizing intervention protocols. The study population should also be expanded to include older adults, cardiovascular disease patients, and other chronic disease groups to evaluate BFR’s safety and applicability in these populations. Additionally, large-scale, multicenter randomized controlled trials should assess the long-term efficacy and safety of BFR. These studies should incorporate patient-reported outcomes such as quality of life and functional indicators to increase clinical relevance.Moreover, exploring the synergistic effects of BFR with other rehabilitation methods (e.g., physical therapy, electrical stimulation) and developing intelligent devices for dynamic training parameter adjustments could enable personalized and precise rehabilitation solutions. These advancements will provide a stronger scientific foundation for the widespread application of BFR in rehabilitation medicine.

## Supporting information

S1 File PRISMA 2020 checklist for systematic reviews. (PDF)

S2 Table Risk of bias and quality assessment summary of the included studies. (XLSX)

S3 Table List of included and excluded studies with specific reasons for inclusion/exclusion decisions. (XLSX)

S4 File Forest plot data including outcome values (mean, SD, sample size) and detailed numerical breakdown of literature selection steps for the PRISMA flow diagram. (DOCX)

## Abbreviations:

BFR: Blood Flow Restriction
L-BFR: Low-load Blood Flow Restriction
L-CON: Low-load Control
H-RT: High-intensity Resistance Training
1RM: One Repetition Maximum
SMD: Standardized Mean Difference
KOA: Knee Osteoarthritis
TKA: Total Knee Arthroplasty
