# Supplementary material for: The effects of low-load resistance training combined with blood flow restriction on knee rehabilitation in middle-aged and elderly patients: A systematic review and meta-analysis
Source: PLoS One. 2025 Jun 2;20(6):e0323388. doi: 10.1371/journal.pone.0323388 (PMC12129158; doi:10.1371/journal.pone.0323388)
Supplement: S4 File — (DOCX) [file pone.0323388.s004.docx]

| Data of pain score comparison between L - BFR and L - CON | | | | | | |
| --- | --- | --- | --- | --- | --- | --- |
| Subgroup | L-BRF | | | L-CON | | |
|  | Mean | SD | Total | Mean | SD | Total |
| Bryk 2016 | -3.3 | 2.2 | 17 | -2.5 | 1.8 | 17 |
| Ferraz 2016 | -2.9 | 3.12 | 12 | -3.9 | 3.44 | 12 |
| Qinbeiying 2022 | 4.74 | 1.73 | 43 | 4.93 | 1.7 | 42 |
| Segal 2014 | 2 | 2.8 | 19 | 1.8 | 2.7 | 21 |
| Segal 2015 | 2.9 | 10 | 19 | 5.6 | 11.7 | 22 |

1. **Corresponding to the specific data in the forest plot**

| Data of muscle strength comparison between L - BFR and L - CON | | | | | | |
| --- | --- | --- | --- | --- | --- | --- |
| Subgroup | L-BRF | | | L-CON | | |
|  | Mean | SD | Total | Mean | SD | Total |
| Bryk 2016 | 16.8 | 10.3 | 17 | 9.4 | 8.3 | 17 |
| Ferraz 2016 | 31.69 | 15.68 | 12 | 9.78 | 8.35 | 12 |
| Qinbeiying 2022 | 1.01 | 0.2 | 43 | 0.7 | 0.19 | 42 |
| Segal 2015 | 18.3 | 6.16 | 38 | 14.65 | 7.53 | 43 |
| Tianxiuling | 3.09 | 0.65 | 50 | 2.45 | 0.39 | 50 |

| Data of muscle strength comparison between L - BFR and H - RT | | | | | | |
| --- | --- | --- | --- | --- | --- | --- |
| Subgroup | L-BRF | | | H-RT | | |
|  | Mean | SD | Total | Mean | SD | Total |
| Bryk 2016 | 16.8 | 10.3 | 17 | 9.4 | 8.3 | 17 |
| Ferraz 2016 | 31.69 | 15.68 | 12 | 44.74 | 12.52 | 10 |
| Harper 2019 | 52.81 | 12.31 | 16 | 54.29 | 9.36 | 19 |
| Reynaldo 2019 | 25.42 | 7.0 | 16 | 27.49 | 5.6 | 16 |

| Data of muscle pain comparison between L - BFR and H - RT | | | | | | |
| --- | --- | --- | --- | --- | --- | --- |
| Subgroup | L-BRF | | | H-RT | | |
|  | Mean | SD | Total | Mean | SD | Total |
| Bryk 2016 | -3.3 | 2.2 | 17 | 2.5 | 1.8 | 17 |
| Ferraz 2016 | -2.9 | 3.12 | 12 | -2.6 | 2.76 | 10 |
| Harper 2019 | -0.69 | 0.91 | 16 | -0.92 | 0.97 | 19 |
| Reynaldo 2019 | -12.69 | 16.35 | 16 | -1.79 | 7.42 | 16 |

| Data of muscle strength comparison between pre - operative L - BFR and L - CON | | | | | | |
| --- | --- | --- | --- | --- | --- | --- |
| Subgroup | L-BRF | | | L-CON | | |
|  | Mean | SD | Total | Mean | SD | Total |
| Franz 2022 | 30.8 | 11.1 | 10 | 15.3 | 9.4 | 10 |
| Jørgensen 2024 | -0.2 | 0.96 | 42 | -0.1 | 0.99 | 44 |
| SWANK 2011 | 60.0 | 5.4 | 36 | 50.7 | 5.5 | 35 |
| Zhujinyu 2024 | 11.86 | 3.08 | 32 | 9.32 | 1.84 | 32 |

| Data of muscle pain comparison between pre - operative L - BFR and L - CON | | | | | | |
| --- | --- | --- | --- | --- | --- | --- |
| Subgroup | L-BRF | | | L-CON | | |
|  | Mean | SD | Total | Mean | SD | Total |
| Franz 2022 | 76.01 | 3.96 | 10 | 70.78 | 7.76 | 10 |
| Jørgensen 2024 | 31.6 | 20.47 | 42 | 41.2 | 20.14 | 44 |
| SWANK 2011 | 5.7 | 0.38 | 36 | 4.9 | 0.42 | 35 |

1. **Literature Selection Process**

| Stage | Description | Number | Notes |
| --- | --- | --- | --- |
| Identification | Records identified from Databases | 365 |  |
| Identification | Records identified from Registers | 64 |  |
| Identification | Records removed before screening (Duplicate, Ineligible, Other reasons) | 134 (Duplicate) + 109 (Ineligible) + 67 (Other reasons) | Duplicate records removed, records marked as ineligible, records removed for other reasons |
| Screening | Records screened | 119 |  |
| Screening | Reports sought for retrieval | 58 |  |
| Screening | Reports assessed for eligibility | 20 |  |
| Screening | Reports excluded (Reviews, commentaries, animal experiments) | 61 (Reviews, commentaries, animal experiments) | Inconsistent interventions or treatments, Insufficient data, Outcome measures inconsistent |
| Screening | Reports excluded due to poor experimental design | 38 |  |
| Included | Studies included in review | 12 |  |
